# Kinesiophobia and related influencing factors in stroke patients with hemiplegia: a cross-sectional study and parallel mediation analysis

**DOI:** 10.3389/fpubh.2026.1795139

**Published:** 2026-05-12

**Authors:** Xing Chen, Yinyin Fan, Quanli Liu, Shuijuan Wang

**Affiliations:** 1 The First People's Hospital of Jintan, Changzhou, China; 2 Nantong University, Nantong, China

**Keywords:** anxiety and depressive symptoms, kinesiophobia, mediation effect, pain catastrophizing, self-efficacy, stroke

## Abstract

**Objective:**

This study aimed to investigate the mediating role of pain catastrophizing and self-efficacy in the association between anxiety and depressive symptoms and kinesiophobia among stroke patients with hemiplegia.

**Methods:**

A cross-sectional study was conducted between March 2023 and September 2024 in a tertiary hospital in Jiangsu Province, China. A total of 260 stroke patients with hemiplegia were enrolled and completed assessments including a demographic questionnaire, the Tampa Scale for Kinesiophobia (TSK), the Pain Catastrophizing Scale (PCS), the Hospital Anxiety and Depression Scale (HADS), and the Self-Efficacy for Exercise Scale (SEE). Multiple linear regression was performed to identify independent factors associated with kinesiophobia. Parallel mediation analysis was applied to examine whether psychoemotional factors mediated the relationship with kinesiophobia.

**Results:**

Multivariate linear regression revealed that age (*β* = −0.054, *p* = 0.027), fall history (*β* = 0.082, *p* = 0.002), pain catastrophizing (*β* = 0.569, *p* < 0.001), self-efficacy (*β* = −0.267, *p* < 0.001), and anxiety and depressive symptoms (*β* = 0.115, *p* = 0.004) were significantly associated with kinesiophobia in stroke patients with hemiplegia. Anxiety and depressive symptoms exhibited a significant total effect on kinesiophobia (total effect = 0.788, *p* < 0.001), with pain catastrophizing and self-efficacy acting as mediators along two separate pathways.

**Conclusion:**

Kinesiophobia in stroke patients with hemiplegia was associated with pain catastrophizing, anxiety and depressive symptoms, self-efficacy, and fall history. The structural equation modeling (SEM) revealed that negative emotions may increase kinesiophobia through greater pain catastrophizing and reduced self-efficacy. Thus, interventions aimed at building self-efficacy and mitigating pain catastrophizing may effectively decrease kinesiophobia and facilitate recovery.

## 1 Introduction

Stroke is a major global health challenge. According to the 2019 Global Burden of Disease (GBD) study, it remained the second-leading cause of death worldwide (1). In China, stroke is not only the primary cause of adult disability and mortality, but also contributed to 3.94 million new cases in 2019 (2). Beyond its profound impact on individual patients, the condition places a substantial burden on families and healthcare systems (3).

Approximately 80% of stroke patients experience motor or linguistic impairments, with post-stroke hemiparesis being the most prevalent and severe (4, 5). These patients typically present with upper motor neuron syndrome, characterized by limb paralysis, hypertonia, hyperreflexia, and sensory nerve damage. These conditions not only severely impact the patients’ physical and psychological wellbeing but also place a substantial burden on both the survivors and society at large (5, 6).

Stroke rehabilitation is an integrative process that employs medical, social, and educational strategies to maximize a patient’s physical, psychological, and socio-professional recovery within their physiological and environmental limits (7). It is considered essential for improving function and reducing symptoms in hemiplegic patients (8). Despite this, studies show that about 40% of survivors are left with slight to moderate deficits (9). A primary obstacle to effective recovery is patient inactivity, which is often rooted in the psychological barrier of kinesiophobia (10).

First coined by Kori in 1990, kinesiophobia is defined as an irrational and debilitating fear of physical movement, arising from a feeling of vulnerability to painful injury or re-injury (11). Vlaeyen introduced the cognitive fear-avoidance model based on previous research. It is hypothesized that pain catastrophizing and kinesiophobia represent a magnifying focus of attention aimed at somatosensory experiences, inflating this perception in terms of a pain-related exaggeration of anxious expectation of the pain getting worse and worse. Furthermore, the fear-avoidance model considers psychosomatic and psychiatric diseases, such as depression or panic disorder, to be conditions that typically enhance this focus on pain signals (12). Vlaeyen’s perspective states that kinesiophobia can result in avoidance behavior, which over time causes incapacity, desperation, impairment, and a patient trapped in a vicious circle of increased pain fears, worsening incapacity, and more distress (13). Therefore, improving the rehabilitative function of stroke patients with hemiplegia by addressing their kinesiophobia is vital from both a clinical and social perspective.

Stroke-specific evidence has increasingly supported the clinical relevance of kinesiophobia in rehabilitation. Fear-avoidance behavior has been shown to predict community reintegration among community-dwelling stroke survivors (14). In addition, kinesiophobia in stroke patients has been associated with advanced age, longer disease duration, and greater stroke severity (15). More recent evidence in stroke patients with hemiplegia has also linked kinesiophobia to history of falls, anxiety and depressive symptoms, and low exercise self-efficacy (16). These findings suggest that kinesiophobia is an important barrier to post-stroke rehabilitation and may be shaped by both general psychosocial processes and stroke-specific clinical factors. Nevertheless, the potential mechanisms linking anxiety and depressive symptoms to kinesiophobia in stroke patients with hemiplegia remain insufficiently understood. Therefore, this study aimed to explore that relationship and to further examine the mediating roles of pain catastrophizing and self-efficacy.

Although the fear-avoidance model was originally developed in chronic musculoskeletal pain populations (17), its core processes may still provide a useful conceptual framework for understanding kinesiophobia in stroke rehabilitation. However, in stroke patients with hemiplegia, the perceived threat may arise not only from pain or fear of reinjury, but also from fear of falling, impaired balance, postural instability, motor weakness, and uncertainty regarding movement safety. Maladaptive psychosocial thought patterns emerge when pain is perceived as a threat, which can exacerbate pain and lead the patient down a path toward despair and disability (18). Functional disuse and poor psychological health are correlated in both directions. Restricted mobility results from poor psychological health, and dysfunction and stiffness result from restricted movement, which in turn serves to confirm the initial beliefs (19). This dynamic process is not conducive to a successful recovery (20). In light of this interaction, the research on the potential mechanism in this path is also significant.

In addition to the direct impact of anxiety and depressive symptoms on the kinesiophobia of stroke patients, the potential mechanism in this pathway is also worthy. Pain catastrophizing refers to a predisposition to ruminate, magnify, and feel helpless about pain (21). Studies have established pain catastrophizing as a contributor to kinesiophobia. For example, one-year follow-up data revealed that pain catastrophizing scores were a significant predictor of kinesiophobia. This link may be explained by patients’ maladaptive pain cognitions, wherein they magnify the threat of pain—an irrational belief that hinders rehabilitation (22). Additionally, a large number of empirical research studies have revealed that pain catastrophizing and other general negative emotional variables, including depression, anxiety, neuroticism, and negative affect, consistently share a substantial amount of variance (23). Considering the association of pain catastrophizing with kinesiophobia and anxiety and depressive symptoms, involvement in pain catastrophizing among stroke patients with hemiplegia may worsen negative emotion symptoms and further exacerbate kinesiophobia. Thus, one of the aims of this study is to verify whether pain catastrophizing has a mediating effect between kinesiophobia and anxiety and depressive symptoms in stroke patients with hemiplegia.

Previous studies supported the notion that greater self-efficacy for exercise had less kinesiophobia. For example, a clinical trial noted that every 10-unit increase in SEE score was linked to 0.87 times the likelihood of kinesiophobia and that physical activity and behavioral interventions can improve negative emotional symptoms (24). Self-efficacy describes a person’s confidence to carry out a specific activity or task (25). Various factors affect self-efficacy, including socio-demographic and emotion-related variables (26). Therefore, self-efficacy might be a potential mediator between kinesiophobia and anxiety and depressive symptoms among stroke patients with hemiplegia.

Hence, this investigation evaluated kinesiophobia among stroke patients with hemiplegia via questionnaires, while incorporating psychological assessments for pain catastrophizing and self-efficacy to explore the connection between kinesiophobia and anxiety and depressive symptoms, alongside identifying kinesiophobia risk factors.

## 2 Materials and methods

### 2.1 Participants

This study is cross-sectional. From March 2023 to September 2024, participants were chosen from a tertiary hospital in Jiangsu Province. During the hospitalization for the stroke, all participants underwent evaluation. In this study, convenience sampling was employed. No formal *a priori* sample size calculation was performed, and the final sample size was determined by the number of eligible patients consecutively recruited during the study period. The inclusion criteria were adult patients that: (a) stroke survivors who met the diagnostic criteria for cerebrovascular disease and were diagnosed with cerebral infarction or cerebral hemorrhage by head computed tomography or magnetic resonance imaging; (b) were aged ≥18 years; (c) unilateral limb hemiparesis, upper limb Brunnstrom II–IV stages; (d) the Montreal Cognitive Assessment (MoCA) score>26; and (e) provided written informed consent. We excluded those that: (a) Congenital limb dysfunction; (b) organic diseases of the heart, liver, kidney, and other vital organs; (c) lacked emotional support and cooperation due to co-occurring mental disorders.

### 2.2 Data collection

This study has been approved by the Ethics Committee (2023-K063-01), and participants were gathered from the wards. The survey was conducted the day before patient discharge. Before the official investigation, the researcher received standard training in data collection techniques. Face-to-face interviews were used to collect data. At first, the investigators briefed the qualified subjects on the aim of the research; those who agreed to take part completed an informed consent form to take part in the official study. Before participants independently completed the questionnaire, the researchers explained the standard operating procedures for the data-gathering tool. If participants had any questions or concerns, the researchers were there to address them. The researcher read each question on the questionnaire and noted the participants’ answers if they could not finish it independently. Around 20–25 min were needed to finish the questionnaire. To prevent a reevaluation, completed questionnaires were promptly reviewed for deficiencies.

### 2.3 Measures

#### 2.3.1 Demographic characteristics

The general demography and disease data collection table specifically involve age, gender, education level, state of life, financial status, accompanying diseases, type of stroke, residence, NIHSS score, Body Mass Index (BMI), and any history of falls.

#### 2.3.2 Kinesiophobia

Tampa Scale of Kinesiophobia (TSK) The 17-item Tampa Scale for Kinesiophobia (TSK-17) was used to assess kinesiophobia. Each item is rated on a 4-point Likert scale, and the total score ranges from 17 to 68, with higher scores indicating greater kinesiophobia. A score of >37 was considered indicative of high kinesiophobia. The Chinese version has been validated in Chinese patients with chronic pain (27). In our study, the Cronbach’s *α* coefficient for this scale was 0.748.

#### 2.3.3 Pain catastrophizing

Pain Catastrophizing Scale (PCS) The scale consists of three dimensions: contemplation, exaggeration, and helplessness. The range was from 0 to 52, and higher scores on the scale indicate higher levels of catastrophizing pain (28). The Cronbach’s *α* coefficient for this scale in our study was 0.955.

#### 2.3.4 Self-efficacy

Self-Efficacy for Exercise Scale (SEE): The scale has nine items and is not dimensional. Higher scores indicate a higher degree of exercise self-efficacy (29). In our study, the Cronbach’s *α* coefficient for this scale was 0.776.

#### 2.3.5 Anxiety and depressive symptoms

Anxiety and depressive symptoms were assessed using the validated Chinese version of the Hospital Anxiety and Depression Scale (HADS) (30). The HADS consists of two subscales, namely anxiety and depression. Higher scores indicate greater anxiety and depressive symptom burden. In this study, the HADS score was used to reflect psychological distress characterized by anxiety and depressive symptoms, rather than the full spectrum of negative emotions or a formal clinical diagnosis. The Cronbach’s *α* coefficient for this scale in our study was 0.745.

### 2.4 Statistical analyses

Statistical analyses were performed using IBM SPSS 25.0 and AMOS 25.0.

(1) Descriptive statistics: Measurement data conforming to a normal distribution were expressed as mean ± standard deviation (X̅ ± SD), while non-normal distribution data were presented as median (25th and 75th percentiles) or frequency (percentage). (2) Univariate analysis: Spearman’s correlation was used to assess associations between kinesiophobia and psychological scales. Group differences in kinesiophobia scores were tested with the Mann–Whitney *U* or Kruskal–Wallis *H* test. (3) Multivariate analysis: Variables with *p* < 0.05 in univariate analyses were entered into a multiple linear regression to identify factors associated with kinesiophobia. (4) Mediation analysis: A parallel mediation model tested whether psychoemotional factors mediated effects on kinesiophobia, using 5,000 bootstrap samples with 95% percentile confidence intervals.

## 3 Results

### 3.1 Patient characteristics and results of univariate analysis on the factors of kinesiophobia in stroke patients with hemiplegia

A total of 260 patients were included, aged 65 (55,72) years old; among them, 171 cases (65.8%) were male, and 89 cases (34.2%) were female; education level was a junior secondary school and below in 36 cases (13.8%), high school in 209 patients (80.4%), and college and above in 15 cases (5.8%); the type of stroke was ischemic stroke in 138 patients (53.1%) and hemorrhagic stroke in 122 cases (46.9%). Other socio-demographic descriptions are detailed in Table 1. Univariate analysis indicated that gender, age, stroke type, and history of falls were significantly associated with kinesiophobia, whereas NIHSS was not significantly associated with kinesiophobia.

**Table 1:** Demographic and clinical characteristics and results of univariate analysis on the factors of kinesiophobia in stroke patients with hemiplegia (*N* = 260).

Variables	Socio-demographic characteristics	*N* (%)	*r/Z / H*	*p*
Gender^b^	Male	171 (65.8%)	−2.298	**0.022**
	Female	89 (34.2%)		
Age^a^		65 (55,72)	−0.225	**<0.001**
NIHSS		2.09 ± 1.73	−0.064	0.302
BMI^c^	<18.5	6 (2.3%)	4.286	0.232
	18.5–23.9	100 (38.5%)		
	24–27.9	116 (44.6%)		
	> = 28	38 (14.6%)		
State of life^c^	Living with spouse	172 (66.2%)	2.278	0.320
	Living alone	9 (3.5%)		
	Living with children	79 (30.4%)		
Education level^c^	Low	36 (13.8%)	1.411	0.494
	Intermediate	209 (80.4%)		
	High	15 (5.8%)		
Residence^b^	City	142 (54.6%)	−1.651	0.099
	Village	118 (45.4%)		
Financial status	Fully available	124 (47.7%)	2.590	0.274
	Just barely	111 (42.7%)		
	Difficulty	25 (9.6%)		
Accompanying diseases^b^	Yes	110 (42.3%)		
	No	150 (57.7%)		
Type of stroke^b^	Ischemic stroke	138 (53.1%)	−7.202	**<0.001**
	Hemorrhagic stroke	122 (46.9%)		
History of falls^b^	Yes	71 (27.3%)	−6.901	**<0.001**
	No	189 (72.7%)		

Values are presented as number and the median (25th and 75th percentiles).

Variables in bold are those considered statistically significant. BMI, body mass index.

a Values are analyzed by Spearman correlation analysis (*r*).

b Values are analyzed by Mann–Whitney *U* test (*Z*).

c Values are analyzed by Kruskal–Wallis *H* test (*H*).

### 3.2 Multivariate linear regression model of the factors associated with kinesiophobia in stroke patients with hemiplegia

Table 2 displays the results of a multiple linear regression analysis identifying predictors of kinesiophobia in stroke patients with hemiplegia. The model showed no multicollinearity (VIF range: 1.055 ~ 2.868) and independent residuals (Durbin Watson = 1.825). These five predictors collectively explained 85.50% of the variance in kinesiophobia. Significant factors included age (*β* = −0.054, *p* = 0.027), history of falls (*β* = 0.082, *p* = 0.002), pain catastrophizing (*β* = 0.569, *p* < 0.001), self-efficacy (*β* = −0.267, *p* < 0.001), and negative emotion (*β* = 0.115, *p* = 0.004). All variables remained significant after false discovery rate (FDR) correction.

**Table 2:** Multivariate linear regression model of the factors associated with kinesiophobia in stroke patients with hemiplegia (*N* = 260).

Variables	*B* (SE)	*β*	*t*	*p*-value	*P*-FDR	(95%CI)
Age	−0.038 (0.017)	−0.054	−2.225	0.027	0.027	(−0.072, −0.004)
History of falls	1.591 (0.504)	0.082	3.158	0.002	0.003	(0.599, 2.584)
Pain catastrophizing	0.410 (0.029)	0.569	14.231	<0.001	<0.001	(0.353, 0.467)
Self-efficacy	−0.107 (0.015)	−0.267	−7.176	<0.001	<0.001	(−0.137, −0.078)
Anxiety and depressive symptoms	0.117 (0.04)	0.115	2.945	0.004	0.005	(0.039, 0.195)

Model summary: *p* < 0.001, adjusted *R*^2^ = 85.8%. SE standard error; FDR, false discovery rate.

### 3.3 Structural equation modeling (SEM) of kinesiophobia, anxiety and depressive symptoms, pain catastrophizing, and self-efficacy

**Figure 1:**
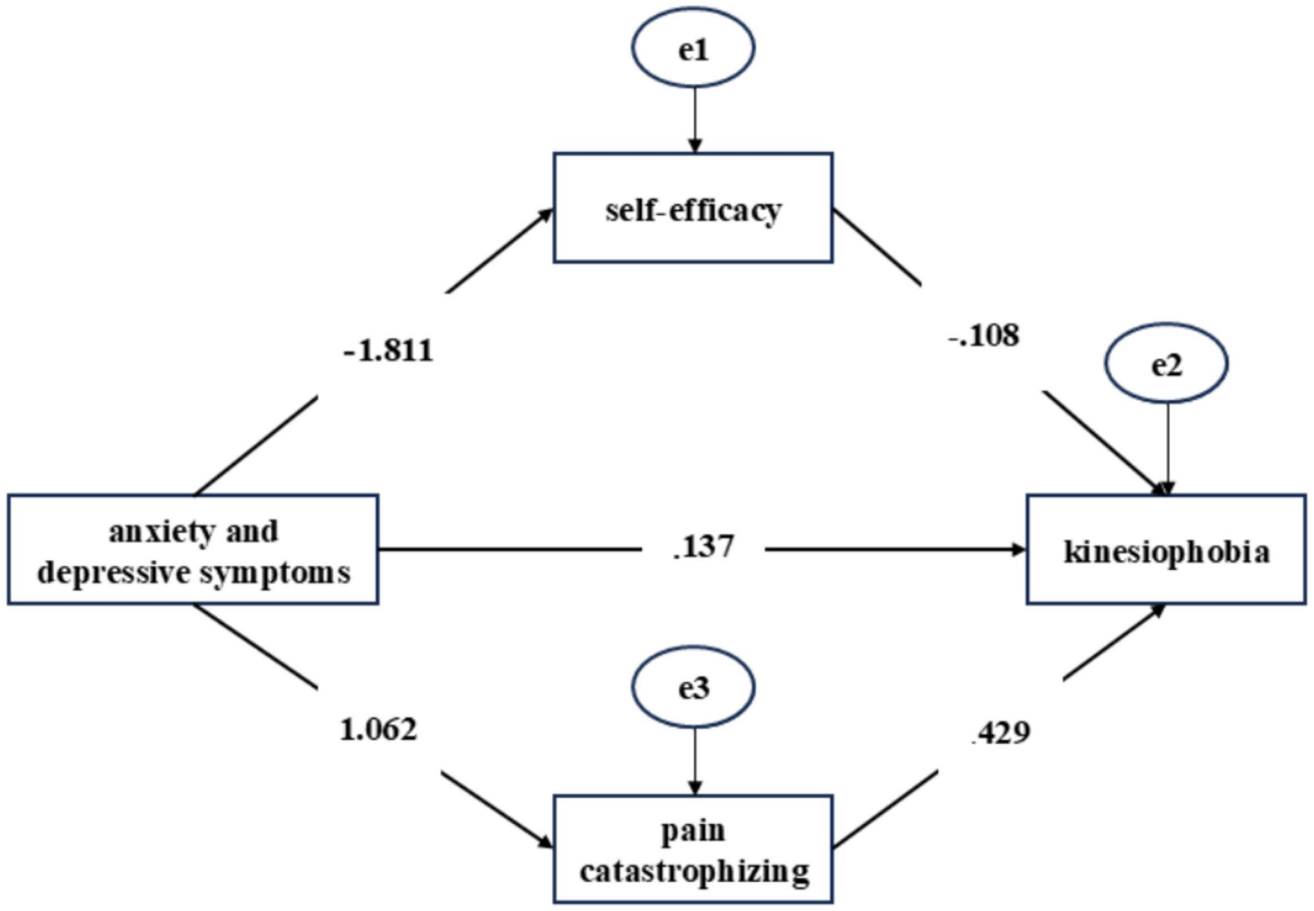
Structural equation modeling (SEM) of the kinesiophobia, anxiety and depressive symptoms, pain catastrophizing, and self-efficacy.

Table 3 presents correlation analyses of psychological characteristics in stroke patients with hemiplegia. Kinesiophobia showed significant correlations with negative emotion (*r* = 0.747, *p* < 0.01), pain catastrophizing (*r* = 0.855, *p* < 0.01), and self-efficacy (*r* = −0.753, *p* < 0.01). Structural equation modeling (SEM) confirmed mediating roles of pain catastrophizing and self-efficacy between negative emotion and kinesiophobia. The model fit well (*χ*^2^/df = 2.253, RMSEA = 0.043, GFI = 0.920, CFI = 0.948, NFI = 0.948). Pathway analysis indicated a significant total effect of anxiety and depressive symptoms on kinesiophobia (effect = 0.788, *p* < 0.001). The direct effect of anxiety and depressive symptoms on kinesiophobia was also retained in the mediation model (c′ = 0.137), in addition to the two indirect paths through self-efficacy and pain catastrophizing, with a significant difference in the magnitude of the two indirect effects (Table 4 and Figure 1).

**Table 3:** Psychological characteristics of stroke patients with hemiplegia, and the connection with kinesiophobia (*N* = 260).

Variables	Kinesiophobia	Pain catastrophizing	Self-efficacy	Anxiety and depressive symptoms
Kinesiophobia	1			
Pain catastrophizing	0.855**	1		
Self-efficacy	−0.753**	−0.681**	1	
Anxiety and depressive symptoms	0.747**	0.728**	−0.701**	1

** *p* < 0.01.

**Table 4:** The results of direct, indirect, and total effects on mediation analysis.

Effects	Standardized estimator	LLCI–ULCI	*p-*value
Direct effects			
Anxiety and depressive symptoms → kinesiophobia	0.137	0.058 ~ 0.220	0.002
Indirect effects			
Ind1	0.195	0.139 ~ 0.270	<0.001
Ind2	0.456	0.370 ~ 0.553	<0.001
Total effects			
Anxiety and depressive symptoms → kinesiophobia	0.788	0.719 ~ 0.858	<0.001
Diff	−0.261	−0.388 to −0.121	0.001

LLCI, low-limit confidence interval; ULCI, upper-limit confidence interval.

Model summary: *χ*^2^/df = 2.253, RMSEA = 0.043, GFI = 0.920, CFI = 0.948, NFI = 0.948.

## 4 Discussion

In this study, stroke patients with hemiplegia exhibited a median kinesiophobia score of 35.00 (IQR: 32.00 ~ 47.00), indicating a level comparable to or potentially higher than that of other stroke populations. Significant predictors identified through multiple linear regression included age, history of falls, pain catastrophizing, self-efficacy, and negative emotion. However, the relatively high explained variance should be interpreted cautiously, as the psychological variables were strongly intercorrelated in this study. Although the VIF values did not indicate severe statistical multicollinearity, substantial shared variance or conceptual overlap among these self-report constructs cannot be fully excluded. These results suggest that addressing adverse psychological factors—particularly pain catastrophizing, negative emotions, and low self-efficacy—through targeted psychological and psychosocial interventions may help reduce kinesiophobia in this patient group.

This study examined various factors influencing kinesiophobia among stroke patients with hemiplegia from diverse perspectives. From a sociodemographic standpoint, age emerged as a risk factor of kinesiophobia, consistent with previous research (7). As patients with hemiplegia due to stroke age, their physical functions decline, muscle strength weakens, and they become intolerant to exercise, which can easily lead to avoidance behaviors and fear of movement (10). At the same time, older adults have more rigid cognitive patterns and are less receptive to new things. This suggests that medical staff should first assess the patient’s age when intervening in kinesiophobia and then implement staged and individualized intervention strategies accordingly. Regarding clinical characteristics, the severity of kinesiophobia varied among history of falls, The more times a patient falls, the more obvious their kinesiophobia becomes, which is consistent with the previous research (31). Falling experiences are prone to trigger psychological fear, which in turn leads to avoidance behavior and hinders social participation (32). Therefore, medical staff should use fall risk assessment tools to promptly identify high-risk factors and develop targeted exercise programs and protective measures to prevent falls and the negative cycle they cause, and alleviate patients’ kinesiophobia.

Low self-efficacy is a significant contributor to kinesiophobia in hemiplegic stroke patients, aligning with existing findings (22). Exercise self-efficacy was defined as an individual’s confidence in setting and achieving exercise goals—plays a key role in motivating physical activity (33). Patients with higher self-efficacy tend to recognize the benefits of exercise more clearly, demonstrate greater willingness to engage in it, and consequently experience less kinesiophobia, leading to more positive health behaviors. Supporting this, Woby et al. (20) confirmed that improving self-efficacy can effectively reduce fear of pain. Thus, healthcare providers should clearly communicate to patients and their families the importance of gradually increasing activity levels. Encouraging early rehabilitation in a safe setting can help enhance self-efficacy and facilitate recovery.

Patients with greater anxiety and depressive symptoms tended to report higher kinesiophobia. In the present study, this construct was operationalized using the HADS and should therefore be interpreted as symptom burden of anxiety and depression, rather than the broader domain of all negative emotions. During hospitalization, patients are prone to negative emotions such as anxiety due to illness or trauma, insufficient understanding of their condition and poor communication between doctors and patients, which in turn leads to fear of rehabilitation exercises. Studies have shown that the incidence of anxiety after stroke is 29.3%, and that of depression is 11 to 41%, both are key risk factors for kinesiophobia (34, 35). Patients often develop fear and avoidance due to concerns about prognosis, which is manifested as reluctance to move, affecting functional recovery, increasing psychological burden, reducing quality of life, and increasing the difficulty of nursing (36). Therefore, in clinical practice, patients’ emotional problems should be given due attention, and psychological support and guidance should be provided in a timely manner. Through intervention measures such as emotional regulation and positive guidance, anxiety and depression should be alleviated, kinesophobia reduced, and the recovery process promoted.

Aligned with prior studies emphasizing pain catastrophizing, pain emerged as a key risk factor for kinesophobia among stroke patients with hemiplegia in this study (37). Vogel’s et al. (22) research indicates that patients with pain catastrophes tend to hold a negative view, believing that they are unable to overcome the pain and that the existing medical resources are insufficient to effectively control it. This sense of helplessness leads patients to actively avoid daily physical activities, thereby intensifying the formation of kinesophobia. Eventually, they fall into a vicious cycle of “reduced activity—functional degeneration—aggravated pain”, significantly enhancing their subjective perception of pain. Therefore, clinical workers should actively understand patients’ cognition and experience, help them build confidence in overcoming pain, and reduce their fear of movement. For patients with a tendency towards catastrophic pain, it is recommended to alleviate their fear and anxiety about pain through methods such as psychological therapy and behavioral therapy.

Based on previous studies (18, 19), this study explored the mediating roles of pain catastrophizing and self-efficacy between anxiety and depressive symptoms and kinesiophobia in hemiplegic stroke patients. Using structural equation modeling, we developed a conceptual framework in which anxiety and depressive symptoms were positively associated with kinesiophobia through two parallel mediators, namely pain catastrophizing and self-efficacy. Specifically, greater anxiety and depressive symptoms were associated with higher kinesiophobia through increased pain catastrophizing and reduced self-efficacy. Thus, the pathway through self-efficacy should not be interpreted as suppressive or protective, but rather as another positive indirect pathway linking psychological distress to greater kinesiophobia.

It is worth noting that the theory of pain catastrophizing holds that negative emotions can intensify excessive focus on pain, which in turn leads to fear of movement (38). By reducing pain catastrophizing, it is possible to lower patients’ depression and anxiety, thereby alleviating their fear of movement (39). On the other hand, research findings indicate that patients with high self-efficacy are more inclined to initiate and persist in activities that are beneficial for their recovery (40). However, emotional states such as anxiety and depression can undermine self-efficacy (25). Low self-efficacy is manifested as a lack of confidence in completing tasks while in pain, which becomes an obstacle to recovery and is prone to causing excessive vigilance and long-term avoidance of activities (22). Consequently, therapeutic efforts should target the psychological mechanisms of kinesiophobia, including negative emotions, low self-efficacy, and pain catastrophizing, instead of concentrating only on the manifestation of the kinesiophobia.

## 5 Limitations

This study has several limitations. First, owing to its cross-sectional design, no firm conclusions can be drawn regarding causality among the variables. Although the hypothesized mediation model was specified based on our conceptual framework, the temporal precedence and causal direction among anxiety and depressive symptoms, pain catastrophizing, self-efficacy, and kinesiophobia could not be established. Alternative directional pathways may also exist; therefore, the mediation findings should be interpreted cautiously. Second, participants were recruited from a single tertiary hospital in one region of China using convenience sampling, which may limit the generalizability of the findings. Moreover, all data were collected on the day before discharge, which may have introduced a time-point bias, as patients at this stage may experience distinctive psychological states related to discharge and transition to home. Third, the relatively high proportion of patients with hemorrhagic stroke may have introduced selection bias. In addition, the internal consistency of the SEE was moderate in this sample (Cronbach’s *α* = 0.776), which may have introduced measurement error and warrants caution when interpreting the self-efficacy-related findings. Formal discriminant validity among kinesiophobia, pain catastrophizing, self-efficacy, and anxiety and depressive symptoms was also not examined. Given the strong intercorrelations among these measures, construct overlap and common-method variance may have contributed to the relatively high explained variance. Furthermore, the inclusion criteria restricted the sample to patients with upper limb Brunnstrom stages II–IV and MoCA scores >26, thereby excluding those with very mild or very severe motor impairment and those with cognitive impairment, which may further limit generalizability. The very high correlation between pain catastrophizing and kinesiophobia also suggests possible conceptual or item-content overlap between the PCS and TSK, which should be considered when interpreting the high explained variance. Finally, future studies should adopt longitudinal designs, particularly cross-lagged approaches, and, where feasible, incorporate both pre- and post-stroke assessments to clarify the temporal and reciprocal relationships among pain catastrophizing, self-efficacy, anxiety and depressive symptoms, and kinesiophobia, and to better characterize the impact of stroke on these variables.

## 6 Conclusion

Acknowledging its limitations, this relevant study identified key factors for kinesiophobia in hemiplegic stroke patients: age, fall history, pain catastrophizing, self-efficacy, and negative emotion. SEM revealed that anxiety and depressive symptoms may increase kinesiophobia through exacerbated pain catastrophizing and reduced self-efficacy. Thus, clinicians should assess the role of anxiety and depressive symptoms and provide timely support through tailored psychological strategies like music therapy, art videos, and positive communication.

## Data Availability

The raw data supporting the conclusions of this article will be made available by the authors, without undue reservation.
